# Simulation Study of Seawater Tritium Measurement with Dual-Diameter Scintillating Fiber Arrays Under Variable Radionuclide Interference

**DOI:** 10.3390/s26217066

**Published:** 2026-11-09

**Authors:** Wenyu Cheng, Wenjie Qiu, Xinjie Huang, Husheng Wang, Songjie Shi, Zhirong Guo, Qin Zhang, Jun Yang

**Affiliations:** 1 School of Energy and Power Engineering, Huazhong University of Science and Technology (HUST), Wuhan 430074, China; cwy0806@mail.ustc.edu.cn; 2 Wuhan Second Ship Design and Research Institute, Wuhan 430064, China; m18727166659@163.com (W.Q.); tan116@126.com (X.H.); scuwanghusheng@foxmail.com (H.W.); shisj1020422019@163.com (S.S.); guoeg112@sina.com (Z.G.); 3 Innovation Center for Ocean Nuclear Emergency Radiation Monitoring and Protection Technology, Wuhan 430064, China; 4 Shanghai Institute of Applied Physics, Chinese Academy of Sciences, Shanghai 201800, China

**Keywords:** tritium, seawater, scintillating fiber, dual-diameter array, radionuclide interference, minimum detectable activity concentration

## Abstract

This simulation study uses dual-diameter scintillating fibers and a separately read out reference channel to reduce bias in estimated tritium activity concentration caused by changes in complex seawater backgrounds. The reference signal helps correct background contributions to the tritium estimate. Geant4 responses for seven layouts are coupled to an idealized scintillation–silicon photomultiplier model. Calibration transfer is assessed using 19 literature-matched Sr/Cs pairs in a fixed natural matrix, with composition withheld from the estimator. At baseline optical collection, the 5 mm central cluster reduces absolute concentration bias by 35.5–59.0% across these compositions. For the strongest-interference composition at 100 Bq L^−1^ tritium, the predicted mean decreases from 133.7 to 113.8 Bq L^−1^, and the 4 h root mean squared error decreases from 37.0 to 21.9 Bq L^−1^. The cluster retains 88.8% of the conventional effective response and achieves the lowest calibrated minimum detectable activity concentration (MDA) among the dual-diameter candidates: 47.3–50.6 Bq L^−1^ at 4 h for the calibration and strongest-interference compositions. Its high-interference error advantage persists under alternative readout assumptions. The conventional array retains a lower calibrated MDA and better precision near calibration. These simulations identify a design compromise between tolerance of changing interference and retention of tritium sensitivity.

## 1. Introduction

Tritium (^3^H), the only radioactive isotope among the three naturally occurring hydrogen isotopes, is routinely monitored in nuclear-facility discharges. Marine tritium originates from cosmogenic production, historical weapons testing and nuclear activities, with contributions that vary over space and time [1,2,3]. It commonly occurs as tritiated water and is transported with water. Online monitoring can track concentration changes in effluents and receiving waters during a release, provided that the detector can distinguish tritium signals from interference caused by other radionuclides in the measurement cell.

Nie et al. investigated coastal tritium dispersion in Hangzhou Bay [4], and Qu et al. examined coupled transport in water and the atmosphere [5]. Observations following the Fukushima accident likewise show spatial and temporal variation in marine tritium distributions [6,7]. These findings motivate timely measurements that do not assume proportional changes in tritium and interfering radionuclides.

Liquid scintillation counting provides sensitive laboratory measurements of aqueous tritium [8]. Using electrolytic enrichment, Lin et al. reported a minimum detectable activity concentration (MDA) of 0.10 Bq L^−1^ for 350 mL seawater samples [9]. Zhang et al. developed an enrichment method for 700 mL samples, reported a detection limit of 0.45 tritium units, and applied the method to the East China Sea [10]. These values depend on the sample preparation and statistical conventions used in each study. Preparation, enrichment and counting delay the availability of results. Automated liquid scintillation systems enable online analysis but require reagents, sample conditioning and waste handling [8]. The proposed solid-scintillator design targets reusable in-water monitoring with a separate interference reference. It does not remove the short range of tritium β particles in water. Its intended benefit is therefore complementary to LSC; quantitative detection-limit comparisons require matched sample preparation, volume, counting time and background conditions.

Solid scintillators allow repeated measurements without mixing each sample with a liquid scintillation cocktail. Investigated approaches include granulated flow cells and plastic scintillation microspheres [11,12]. The TRITIUM project developed modular fiber detectors through detector design, transport studies and field installation [13,14]. Sanada et al. combined a pellet flow cell with coincidence measurement, a veto detector and shielding, and used spectral information to identify non-tritium interference qualitatively [15]. Assessing interference is therefore an established requirement for scintillation-based tritium instruments.

Cheng et al. developed a plastic scintillating fiber array with a measured efficiency of 1.6 × 10^−3^ and an unshielded MDA of 3165 Bq L^−1^ over 1600 s [16]. Subsequent studies examined array geometry [17] and simulated geometric optimization together with suppression of external γ rays and cosmic muons [18]. This work motivates the investigation of an internal reference channel. However, the published source, shielding and readout conditions differ from those used here. The reported MDAs provide application context, while the present structural comparison uses a common set of modeled conditions.

Dissolved radionuclides remain inside the measurement cell even when external shielding is effective. Surveys around Chinese coastal nuclear facilities have identified natural ^40^K and ^226^Ra and artificial ^90^Sr and ^137^Cs alongside tritium [19,20]. Coastal ^90^Sr also reflects transport and groundwater inputs [21,22], while observations off Japan show changes in radiostrontium-to-radiocesium ratios [23,24]. Energetic electrons and photon-generated secondary electrons can traverse a thin sensing fiber while depositing an amount of energy within the tritium window. A low-energy counting window alone therefore cannot identify the radionuclide responsible for every accepted event.

The proposed structure combines 1 mm sensing fibers with larger fibers that are read out independently. The fine fibers provide the water–scintillator interface for short-range tritium electrons, whereas the larger fibers collect energy from penetrating radiation. Coincident reference signals reject candidate events, and reference counts are used to estimate the residual background in the sensing window. We test whether this arrangement can reduce composition-induced bias while retaining most of the fine-fiber response when sample composition is withheld from the estimator. Both structures use simultaneous background information, since the conventional array retains its above-endpoint reference.

This simulation study uses dual-diameter scintillating fibers and a separately read out reference channel to reduce bias in estimated tritium activity concentration caused by changes in complex seawater backgrounds. The reference signal provides information about background changes, helping reduce the overestimation of tritium caused by interference under transferred calibration. The structural and estimation concept combines a fine-fiber sensing array, a large-diameter reference cluster, event-level reference selection and reference-channel background correction. Seven layouts share a source library and readout model. Literature-based seawater compositions test calibration transfer as interfering activity concentrations and radionuclide ratios change. The estimator receives channel counts without sample-specific interference composition. Concentration bias and root mean squared error (RMSE) quantify model-predicted measurement error; composition-calibrated MDA characterizes retained counting sensitivity. The comparisons identify a candidate reference diameter and placement, while distinguishing reduced systematic bias from counting precision and detection sensitivity.

## 2. Materials and Methods

### 2.1. Array Geometry

**Figure 1:**
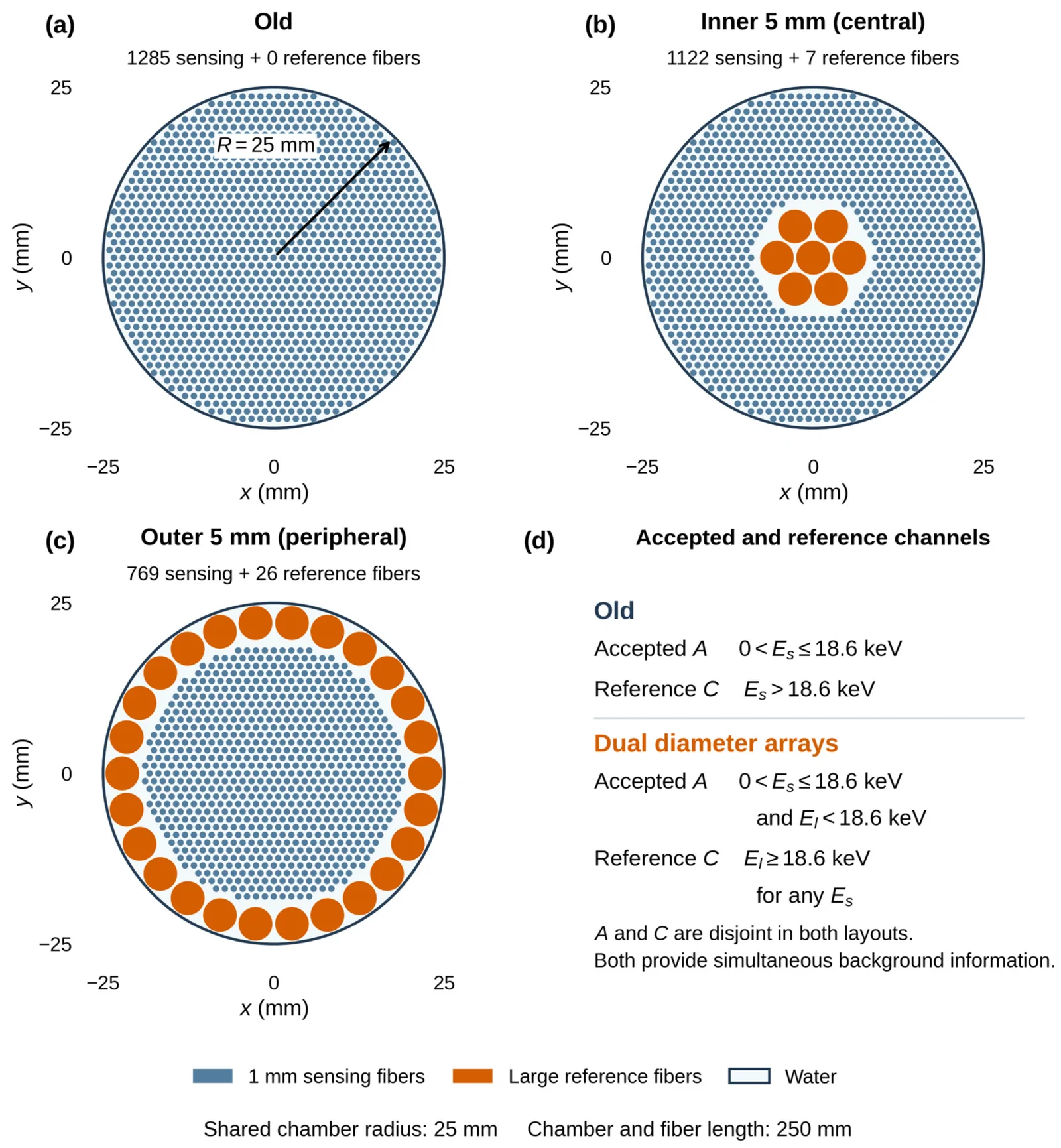
Array geometry and channel definitions. (**a**–**c**) Cross-sections of Old, Inner 5 mm and Outer 5 mm, drawn at the same scale from the supplied placement coordinates. The large reference fibers form a central cluster in (**b**) and a peripheral ring in (**c**). Blue and orange circles denote 1 mm sensing fibers and 5 mm reference fibers, respectively; the remaining chamber area is water. The Inner and Outer layouts contain seven and 26 reference fibers, respectively, so this comparison includes differences in fiber number and sensing area as well as placement. The chamber radius is 25 mm, and the chamber and fibers are 250 mm long. (**d**) Deposited-energy selections for accepted channel A and reference channel C, where E_s_ and E_l_ denote the total energies deposited in the small and large fibers, respectively. The conventional high-energy reference is retained. Both pairs of channels are disjoint and provide simultaneous background information. Primary-photoelectron selections are defined separately in Section 2.3.

Figure 1 compares the conventional array with the Inner and Outer arrays, which contain 5 mm reference fibers at the center and periphery, respectively. The cylindrical measurement chamber has an inner radius of 25 mm; both the chamber and the fibers are 250 mm long. The 1 mm sensing fibers are arranged on a hexagonal lattice with a pitch of 1.3 mm. The conventional array, designated Old, contains 1285 sensing fibers. The Inner arrays contain seven large fibers, whereas the illustrated Outer 5 mm array contains 26. These layouts therefore differ in both reference-fiber placement and number. In the transport model, the fibers are represented as homogeneous polystyrene in direct contact with water. The comparison concerns complete detector layouts within a common chamber; placement, reference-fiber number, sensing area and water volume vary together.

**Figure 2:**
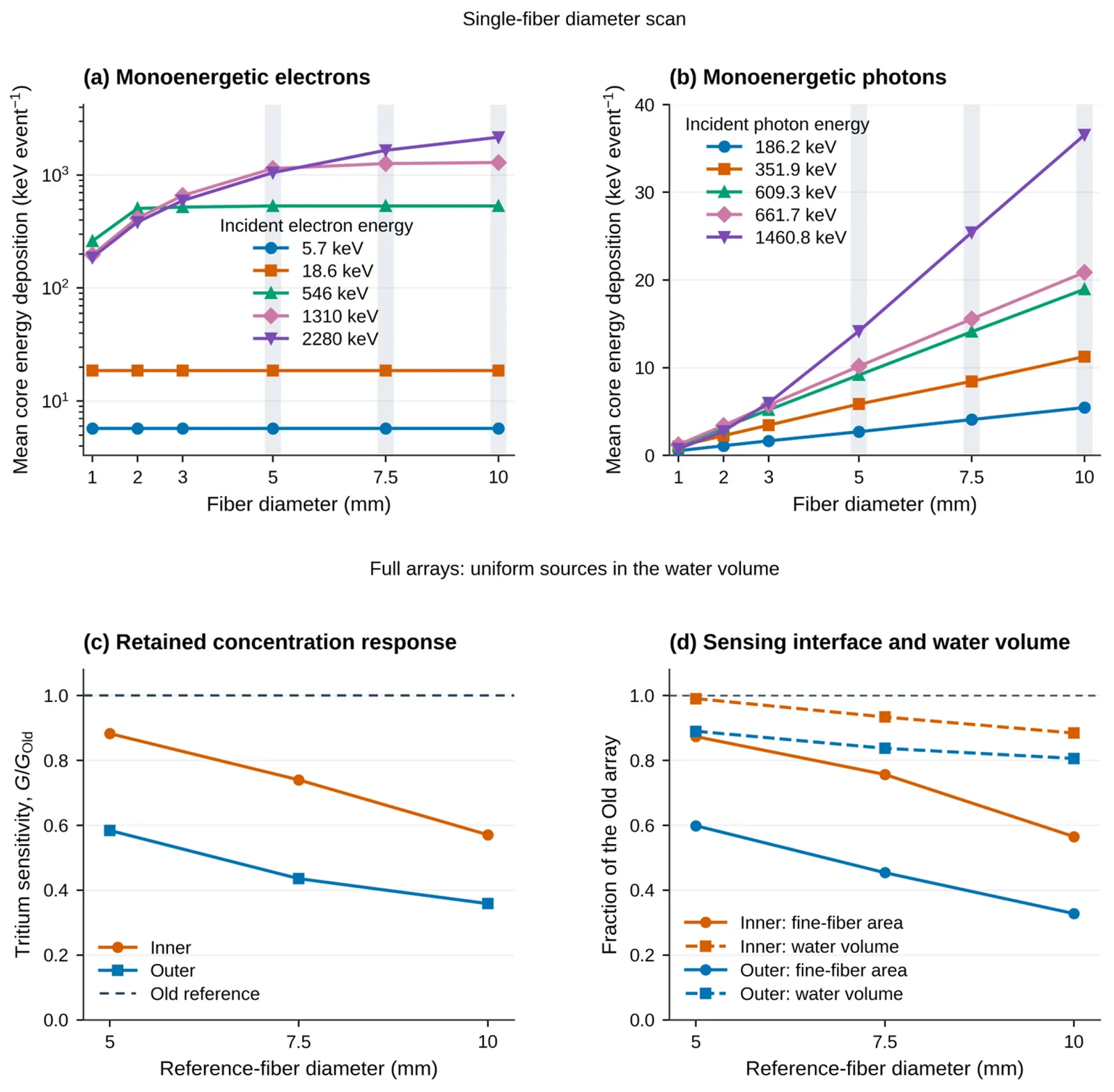
Reference-fiber diameter response and geometric trade-offs in complete arrays. (**a**,**b**) Mean core energy deposition by monoenergetic electrons and photons, respectively, for fiber diameters of 1–10 mm. The responses to 5.7 and 18.6 keV electrons change little with diameter. The 546 keV response approaches a plateau, and the increase in the 1310 keV response slows at 7.5–10 mm, whereas the responses to 2280 keV electrons and photons continue to rise. Shaded bands identify the 5, 7.5 and 10 mm candidates. (**c**) Deposited-energy tritium concentration sensitivity G, normalized to Old. (**d**) Fine-fiber lateral surface area and water volume, each normalized to the corresponding value for Old. Panels (**c**,**d**) describe complete arrays with sources distributed uniformly throughout the water volume; horizontal dashed lines mark unity. Increasing the reference-fiber diameter reduces the retained tritium response, sensing interface and water volume. Inner retains a higher tritium response than Outer at every tested diameter. The Outer layouts contain 26, 16 and 11 reference fibers at diameters of 5, 7.5 and 10 mm, respectively, so their comparison includes changes in both diameter and fiber number. These trends motivate the comparison of the three candidate diameters as a trade-off between higher-energy response and retained tritium sensitivity. Connecting lines are visual guides.

The single-fiber simulations in Figure 2 compare mean energy deposition from monoenergetic electrons and photons at fiber diameters of 1, 2, 3, 5, 7.5 and 10 mm. Energy deposition by tritium-energy electrons changes little with diameter. The response to 546 keV electrons approaches a plateau, and the response to 1310 keV electrons shows diminishing gains at diameters of approximately 7.5–10 mm. In contrast, the responses to 2280 keV electrons and photons continue to increase. Larger reference fibers therefore collect more energy from penetrating radiation, although the gain depends on particle type and energy. The 5, 7.5 and 10 mm candidates span these response regimes. They are evaluated in complete arrays to balance the response to interference against the loss of sensing-fiber area.

All layouts use the same chamber envelope. Introducing large fibers changes the number of sensing fibers, their exposed lateral area and the water volume (Table 1). Comparisons hold activity concentration and counting time constant while using the water volume of each layout. Before optical readout, the tritium count-rate sensitivity per unit concentration is G = V_w_ ε_H_, where V_w_ is the water volume and ε_H_ is the acceptance probability per tritium decay.

### 2.2. Source Compositions and Radiation Transport

The emission library comprises tritium β decay and eight interference cases (Table 2). The ^40^K β and γ branches represent the abundant natural seawater component; ^90^Sr–^90^Y represents penetrating anthropogenic β interference; and the ^137^Cs daughter γ line represents radiocesium-induced secondary electrons. Selected ^226^Ra, ^214^Pb and ^214^Bi γ lines represent natural-series interference documented in coastal surveys [19,20,23]. Each simulated history contains one primary emission and the secondary particles generated during its transport. The yield of each emission is applied once to the corresponding parent activity concentration.

Calibration composition L uses the Ningde regional mean activity concentrations of ^40^K, ^226^Ra, ^90^Sr and ^137^Cs [19]. Transfer samples use literature-based Sr/Cs pairs. All 27 surface samples collected at a depth of 2 m in Table 1 of Casacuberta et al. [23] were screened for matches in the updated cesium dataset using station, local sampling date and nominal depth [25]. Coordinates were converted to decimal degrees and required to agree within 0.001° in each coordinate, consistent with the positional precision of the cesium dataset. This procedure yielded 19 unique pairs: two records lacked corresponding cesium samples, and six failed the coordinate criterion. Selection did not use activity thresholds or detector performance. Cesium activity concentrations were corrected from 6 April 2011 to the respective sampling dates using a half-life of 30.018 y [26], converted from Bq m^−3^ to Bq L^−1^, and rounded to three significant digits. The modeled pairs span 0.0011–0.085 Bq L^−1^ for ^90^Sr and 0.00226–3.46 Bq L^−1^ for ^137^Cs (Table 3).

All 19 pairs are combined with the Yantai natural matrix of 9.99 Bq L^−1 40^K and 0.00930 Bq L^−1 226^Ra [20]. These controlled computational compositions do not reconstruct the original field samples. Identifiers follow the KOK station numbers; H29 denotes station 29, which has the highest cesium input. The near-calibration sample M_ref_ combines the Fuqing natural matrix [19] with 0.0037 Bq L^−1 90^Sr and 0.100 Bq L^−1 137^Cs from a separate cruise sample collected at station 20 on 26 August 2011 [24]. Thus, L, M_ref_ and the 19 station-based compositions define 21 modeled conditions. Five stations summarize the cesium-sorted range at the minimum, nearest-rank quartiles, median and maximum; all stations are retained in the comparisons.

Equal activity concentrations are assigned to the members of ^90^Sr–^90^Y and ^226^Ra–^214^Pb–^214^Bi. These equilibrium assumptions ensure consistent daughter contributions across layouts. All comparisons use the selected emissions in Table 2 rather than complete decay schemes. Specifically, the radiocesium case represents the selected daughter γ line; ^137^Cs β particles and internal-conversion electrons are not included [26].

Radiation transport is simulated in Geant4 11.2.2 [27,28] with G4EmStandardPhysics_option4. Production range cuts are 0.5 μm for electrons and 1 μm for photons and positrons. The modeled seawater comprises 96.5% water and 3.5% NaCl by mass and has a density of 1.025 g cm^−3^. Polystyrene and PTFE use the corresponding Geant4 NIST materials. Primary positions are uniformly distributed throughout the water volume, with isotropic emission directions. Each of the 63 layout–emission combinations contains 10^6^ histories. Histories with no deposited energy remain in the denominator of all per-emission response probabilities, so the small fraction of water decays reaching a fiber is included in the calculated acceptance. The production range cut governs secondary production; it is not a β-particle range or a near-surface restriction on the source volume.

For transport simulations of the complete arrays, electron energies follow the approximate allowed β spectrum

(1) $$f_{\beta }(T)\propto p\;W{\left(T_{0}-T\right)}^{2}F(Z,W)$$

where T is the kinetic energy, T_0_ is the endpoint energy, W = T + m_e_c^2^, and pc = [T(T + 2m_e_c^2^)]^1/2^. The Coulomb factor is F = x/[1 − exp(−x)], where x = 2π α_fs_ Z W/(pc); α_fs_ denotes the fine-structure constant and Z the daughter atomic number. This approximation omits transition-specific forbidden shape factors [29]. Photon cases use the discrete energies and yields listed in Table 2. The same source definitions are used for every geometry. The quantitative effects of the omitted spectral-shape corrections on response and geometry ranking require further source-model refinement.

### 2.3. Deposited Energy and SiPM Response

Let E_s_ and E_l_ denote the energies deposited during one history, summed over all fibers in the small- and large-fiber channels, respectively. The raw small-fiber window is 0 < E_s_ ≤ 18.6 keV. In the dual-diameter arrays, accepted channel A additionally requires E_l_ < 18.6 keV, whereas reference channel C includes every history with E_l_ ≥ 18.6 keV, regardless of E_s_. Old uses the raw window as channel A and E_s_ > 18.6 keV as channel C:

Old: A: 0 < E_s_ ≤ 18.6 keV; C: E_s_ > 18.6 keV

Dual: A: 0 < E_s_ ≤ 18.6 keV, E_l_ < 18.6 keV

(2) Dual: C: E_l_ ≥ 18.6 keV (any E_s_)

For Old, the reference count equals the number of histories with positive small-fiber energy deposition minus the raw-window count. Both pairs of channels are disjoint, and neither deposited-energy reference channel accepts tritium events.

For emission i, P_A,i_ and P_C,i_ are the accepted and reference counts divided by the number of generated histories. The dual-diameter veto fraction is η_v,i_ = 1 − P_A,i_/P_R,i_, where P_R,i_ denotes the raw-window probability. This fraction describes the rejection of coincident candidates and is distinct from the reference-channel efficiency used for statistical correction.

For an energy deposit E in MeV at axial position z, the mean number of primary photoelectrons at end e is

(3) $$\mu_{e}(E,z)=Y_{\gamma }Q\;Ef_{\mathrm{trap},e}\exp(-l_{e}/\Lambda )c_{e}\bar{\mathrm{PDE}}$$

where Yγ denotes the scintillation yield, Q the relative low-energy light yield, f_trap,e_ the fraction guided toward end e, ℓ_e_ the propagation distance, Λ the attenuation length, and c_e_ the effective coupling and photosensor coverage. The overbar indicates spectrum-averaged photon-detection efficiency (PDE). The SP101 parameters follow the optimization study by Cheng et al., published online in 2025 and assigned to the 2026 journal volume [18]: Yγ = 9500 photons MeV^−1^, refractive index n_PS_ = 1.58, emission peak = 423 nm and Λ = 2.10 m. Setting Q = 1 assumes proportional light production. The axial coordinate spans 0 ≤ z ≤ L, where L is the fiber length, ℓ_1_ = z and ℓ_2_ = L − z.

The mean SiPM PDE is set to 0.50 following the optical study [18]; the S14161-4050HS-06 datasheet specifies a PDE of 0.50 at 450 nm and an overvoltage of 2.7 V [30]. Effective coupling and photosensor coverage are represented by c_e_ = 0.50, with c_e_ = 0.75 used to test improved light collection. This factor describes light delivery to the active photosensor area and is separate from PDE. For the bare polystyrene–water interface, n_water_ = 1.34 and f_trap,e_ = (1 − n_water_/n_PS_)/2 under the meridional approximation. Trapping, attenuation, coupling and detection are treated as separate factors, with identical settings and channel cuts across layouts. These coupling factors define common optical assumptions for the structural comparison.

Assuming Poisson scintillation, independent photon detection and low SiPM occupancy, the counts at the two ends are independent conditional on deposited energy and axial position. Requiring at least one primary photoelectron at each end gives the signal-only trigger probability

(4) $$P_{2}(E,z)=(1-e^{-\mu_{1}})(1-e^{-\mu_{2}})$$

**Figure 3:**
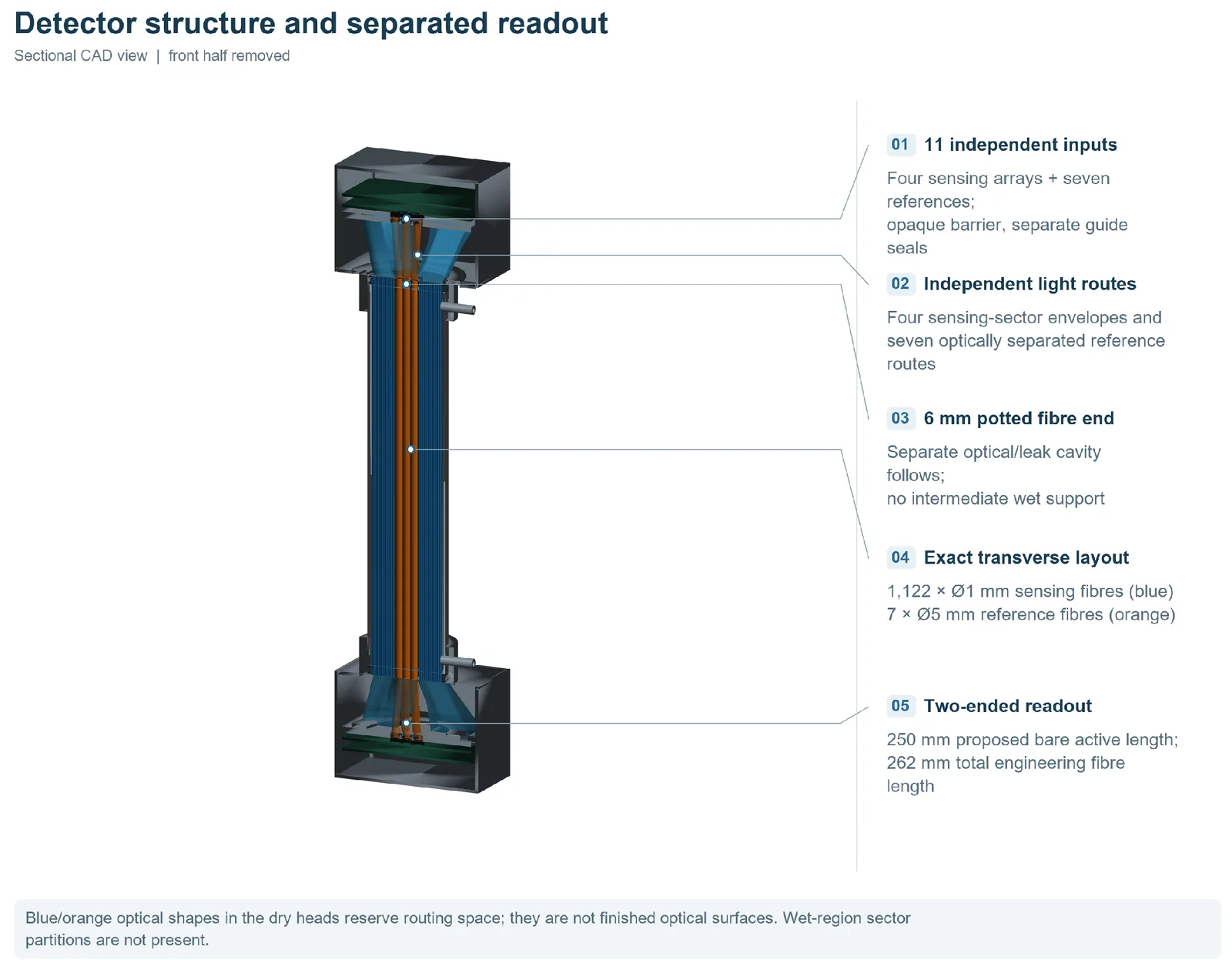
Preliminary sectional design of the Inner 5 mm detector with separate sensing (blue) and reference (orange) routes and two-ended readout. The nominal wet length is 250 mm, with two 6 mm potted ends (262 mm total fiber length). The transport geometry uses 249.98 mm fibers in the 250 mm chamber to avoid coincident numerical boundaries. Dry-head guides and seals are proposed engineering features. The figure is a design schematic; the physical detector is still under development.

For general thresholds, trigger probabilities are calculated from Poisson tails. The distribution of the summed primary-photoelectron count is averaged over deposited energy and axial position. SiPM gain converts avalanches to charge, while optical crosstalk and afterpulsing modify the avalanche distribution [31]. The baseline excludes correlated avalanches and represents a radiation-only primary-photoelectron response without a measured electronics model. The small- and large-fiber groups are optically separated, and each has two modeled end signals. Figure 3 illustrates their proposed physical routing. Device-specific noise, signal summation and coincidence timing will be addressed during prototype characterization; the one-PE-per-end condition is an idealized signal selection.

Let f_i_(x) denote the joint distribution of deposited energy and axial position for emission i and let R(n|x) denote the conditional readout response, where n is the vector of primary-photoelectron counts at the individual ends. For disjoint readout selections Ω_A_ and Ω_C_, the source probabilities are

(5) $${P_{j,i}}^{\mathrm{PE}}=\int f_{i}(x)\sum_{n\in \Omega_{j}}R(n|x)\mathrm{dx}$$

The distribution f_i_ includes all primary histories, including those with zero energy deposition. N_s_ and N_l_ denote the primary-photoelectron counts summed over both ends of the small- and large-fiber channels, respectively. The small-channel window requires 2 ≤ N_s_ ≤ 6 PE, with both ends triggered. For Old, channel C requires N_s_ ≥ 7 PE and triggers at both ends. For the dual-diameter arrays, channel C requires N_l_ ≥ 7 PE and triggers at both large-channel ends; channel A excludes channel C. The upper boundary of 6 PE is obtained by rounding down the baseline mid-fiber mean response at 18.6 keV and is held fixed across layouts and coupling tests. These selections account for interference migration into A and tritium leakage into C.

For readout integration, the probability distribution on the (E_s_, E_l_) grid is obtained by minimizing relative entropy subject to transport constraints [32]:

$$p^{*}={\text{arg min}}_{p}\sum_{m}p_{m}\ln(\frac{p_{m}}{w_{m}})$$

$$A\;p=b,\;\;p\geq 0,\;\;\sum_{m}p_{m}=1$$

(6) $$\sum_{m:E_{s,m}+E_{l,m}\geq u}p_{m}\leq P(T\geq u)(\beta \mathrm{sources})$$

where p_m_ and w_m_ denote cell probabilities and reference weights, respectively, and A p = b encodes the transport tallies. The constraints preserve normalization; zero- and positive-deposit probabilities; accepted and reference fractions; seven joint probabilities P(0 < E_s_ ≤ 18.6 keV, E_l_ < T_k_) with T_k_ = 1, 5, 10, 18.6, 30, 50 and 100 keV; and the first two energy moments. The support satisfies E_s_ + E_l_ ≤ E_0_, where E_0_ is the β endpoint or photon energy. The grid is further constrained by the β-source inequalities P(E_s_ + E_l_ ≥ u) ≤ P(T ≥ u). These constraints retain the available transport information, while the unrecorded energy distribution remains model-dependent. The baseline uses a uniform measure over positive-energy cells with explicit zero-deposit atoms. Its grid contains 74 linear intervals below 18.6 keV and 66 logarithmic intervals above this value and includes all selection thresholds. Half-resolution grids and the reference measure dE/(E + 0.1 keV) are used to test discretization and shape sensitivity. Baseline deposits share a uniformly distributed axial position; independent channel positions are tested separately. This constrained distribution enables readout integration without generating additional Geant4 histories. Differences between assumptions are reported as model sensitivity rather than as confidence intervals. Section 3.5 provides a representative direct-event check of the resulting tritium response probabilities.

### 2.4. Tritium Measurement with Fixed Calibration

In either response domain, the expected background count and the total mean count in channel j are

Bj= t Vw∑iaibiPj,i+trj

(7) μ_j_ = B_j_ + t V_w_ A_H_ P_j,H_, j ∈ {A,C}

where j denotes A or C, t is the acquisition time in seconds, V_w_ is the water volume in litres, a_i_ is the parent activity concentration in Bq L^−1^, and b_i_ is the selected emission yield per decay. A_H_ is the tritium concentration, and P_j,H_ is the corresponding channel response probability; the interference sum excludes tritium. Each calculation consistently uses either deposited-energy probabilities or PE probabilities. Residual rates r_j_ (s^−1^) represent additional backgrounds after channel selection. They are zero in both baseline response domains; specified nonzero rates are used to test background sensitivity.

The background conversion coefficient is κ = B_A_/B_C_. In the absence of additional backgrounds, multiplying all interfering activity concentrations by the same factor leaves κ unchanged. With the calibration coefficient κ_0_ held fixed, the effective tritium concentration sensitivity is G = V_w_(P_A,H_ − κ_0_P_C,H_). For G > 0 and independent Poisson channel counts, the tritium concentration estimator and its counting variance are

$${Â}_{H}=\frac{N_{A}-\kappa_{0}N_{C}}{t\;G}$$

(8) $$\mathrm{Var}({Â}_{H})=\frac{\mu_{A}+{\kappa_{0}}^{2}\mu_{C}}{{\left(t\;G\right)}^{2}}$$

The count difference is not truncated. In the deposited-energy benchmark, P_C,H_ = 0 and G = V_w_ ε_H_. The PE model accounts for tritium leakage into the reference channel in both G and μ_C_. For each layout and readout setting, κ_0_ and G are determined from the modeled L calibration and then held fixed. The estimator uses only the counts in A and C, the acquisition time and these calibration parameters. Within the specified response library, the test compositions, true background ratios and true tritium concentrations define the computational samples and are used to evaluate performance; they are withheld from the estimator. L tests calibration consistency, while the remaining compositions test calibration transfer.

For Inner 5 mm at 100 Bq L^−1^, the water volume of 0.23623 L and energy-domain acceptance of 0.0910% give an accepted tritium count rate of approximately 0.0215 s^−1^ (1.29 min^−1^). The baseline PE acceptance of 0.0333% gives 0.00787 s^−1^ (0.472 min^−1^). The corresponding Old values are 0.0244 and 0.00885 s^−1^. These rates are the product of activity concentration, water volume and the rounded probabilities in Table 1 and Table 4; they exclude interference counts. They differ from the background-corrected concentration response G, which includes tritium leakage into the reference channel.

A change in composition introduces the following systematic bias into the tritium concentration result:

(9) $$\mathrm{Bias}({Â}_{H})=E[{Â}_{H}]-A_{H}=\frac{B_{A}-\kappa_{0}B_{C}}{t\;G}$$

The contributions from changes in the individual sources can be written as

$$\mathrm{Bias}({Â}_{H})=\sum_{i}\Delta a_{i}d_{i}$$

(10) $$d_{i}=\frac{V_{w}b_{i}(P_{A,i}-\kappa_{0}P_{C,i})}{G}$$

where Δa_i_ denotes the change in activity concentration from calibration and d_i_ is the tritium-equivalent response per unit source activity concentration. Branches of the same parent share the same activity-concentration change. Assuming stable readout and residual background, this expression separates changes in radionuclide concentration from geometry-dependent response ratios. Because each source contribution is weighted separately, different Sr/Cs pairs need not produce proportional biases. The expression also provides a consistency check for the multinuclide calculation.

### 2.5. Measurement Error and MDA

MDA is evaluated separately from concentration measurement error under fixed calibration. It denotes the minimum detectable activity concentration in Bq L^−1^, written A_D_ in the equations, rather than the total activity in the cell. We use a nominal one-sided false-positive probability of α = 0.05 and a detection probability of 0.95 [33]. The correct background ratio for each composition defines a composition-calibrated sensitivity benchmark. For independent Poisson counts in the disjoint channels, conditioning on their total gives the binomial upper-tail test [34]:

$$p_{\mathrm{tail}}=\sum_{j=N_{A}}^{n}\frac{n!}{j!(n-j)!}\rho^{j}{\left(1-\rho \right)}^{n-j}$$

(11) $$n=N_{A}+N_{C},\rho =\frac{\kappa }{1+\kappa }$$

A detection is declared when the upper-tail probability is at most 0.05. Averaging the conditional rejection probability over the Poisson distribution of the total count gives the detection power, with omitted tails below 10^−13^; A_D_ is the concentration corresponding to a power of 0.95. The 5% false-positive bound holds for independent Poisson null counts when the applied coefficient equals the true background ratio and is treated as known. The test is also conservative when the applied ratio is higher. When κ_0_ is transferred to another composition, the actual false-positive probability is evaluated using that sample’s true background means rather than assumed to satisfy the nominal bound. These inputs are used only for evaluation and remain withheld from the estimator. Detection-power calculations include tritium leakage into C. Accordingly, composition-calibrated MDA and fixed-calibration performance for an unknown composition are distinct assessment conditions.

Measurement performance is evaluated using the expected measurement result E[Â_H_], absolute bias, counting standard deviation and RMSE. Here, E[Â_H_] is the model-predicted mean of the statistical estimator over repeated acquisitions under the Poisson model, not an experimental reading. Absolute bias is |E[Â_H_] − A_H_|; the direction of the error is stated separately as overestimation or underestimation. A mean closer to the true concentration does not necessarily imply a smaller error in an individual acquisition. RMSE combines squared bias and counting variance:

(12) $$\mathrm{RMSE}=\sqrt{{\mathrm{Bias}({Â}_{H})}^{2}+\mathrm{Var}({Â}_{H})}$$

The primary calculations evaluate measurement performance at true tritium concentrations of 0 and 100 Bq L^−1^ for matched acquisition times of 600, 3600 and 14,400 s (10 min, 1 h and 4 h). A separate 4 h test uses approximately logarithmically spaced concentrations of 30, 100 and 300 Bq L^−1^ while holding interference fixed. These prescribed tritium concentrations vary independently of the literature-based interference concentrations. The time comparison examines when the reduction in squared bias outweighs the increase in counting variance; 4 h is an evaluated interval, not a fitted optimum. Response parameters remain fixed within each comparison, and test-sample truth is not used to adjust selections or calibration.

Wilson intervals characterize individual transport probabilities [35]. Performance rankings are point estimates obtained with response parameters held fixed; in the original 10^6^-history campaign, each layout has hundreds to approximately one thousand accepted tritium histories. Grid refinement, alternative energy and position models, coupling changes, and neighboring integer cuts are used to assess model sensitivity. Calibration uncertainty is distinct from Poisson counting uncertainty [36]. For the deposited-energy estimator, an unbiased estimate of κ with variance u^2^(κ), independent of the sample counts, adds u^2^(κ)(B_C_^2^ + B_C_) to the null numerator variance. In the PE model, κ enters both G and the background correction, so the associated calibration uncertainties are correlated. The reported RMSE includes counting variation and interference-transfer bias under fixed calibration; it does not represent the combined uncertainty associated with all response and calibration assumptions.

Design selection uses the equally weighted mean RMSE across the 19 matched station-based compositions, calculated separately for each readout setting, acquisition time and tritium concentration. The primary ranking uses 100 Bq L^−1^ and 4 h and excludes L and the separate M_ref_ check. This criterion is considered together with retained tritium response and composition-calibrated MDA in L and H29. It is used to select one fixed layout for the tested range; the unknown composition of an individual sample is not used to choose the layout with the lowest error for that sample. This criterion selects among the dual-diameter candidates, with Old retained as the external benchmark. By Equation (12), a lower RMSE requires the reduction in squared bias to exceed the increase in counting variance.

Post-processing was implemented in Python(version 3.14), and figures were generated with Matplotlib 3.10.8 from the input and calculated numerical tables. ChatGPT (OpenAI GPT-6 Astra); accessed September 2026; model version not recorded) assisted in preparing the analysis and plotting code. The calculations follow the equations, selections and reconstruction constraints specified above, and the figures are deterministic visualizations of the underlying numerical data.

## 3. Results

### 3.1. Tritium Measurement Performance Across Seawater Interference Compositions

Under the baseline optical model, at a true tritium concentration of 100 Bq L^−1^, Inner 5 mm reduces absolute bias by 35.5–59.0% across all 19 station-based compositions (Table 5). For S18, the median-cesium composition, the predicted mean concentrations are 102.10 Bq L^−1^ for Old and 100.90 Bq L^−1^ for Inner 5 mm. The corresponding values are 105.77 and 102.40 Bq L^−1^ for the upper-quartile composition S25, and 133.75 and 113.85 Bq L^−1^ for H29. Both layouts predict a mean of 100.00 Bq L^−1^ in L, where the calibration and evaluation compositions are identical. Bias is therefore consistently reduced across the Sr/Cs pairs, although the magnitude of the transfer error varies.

For Inner 10 mm, the predicted mean concentration in H29 is 99.00 Bq L^−1^, closer to the true concentration than the predictions for either Old or Inner 5 mm. However, enlarging the reference cluster displaces sensing fibers and increases counting uncertainty. Bias reduction must therefore be considered together with total measurement error. Across the 19 compositions, the mean absolute bias decreases from 4.30 Bq L^−1^ for Old to 1.79 for Inner 5 mm, whereas the equally weighted mean 4 h RMSE increases from 15.72 to 16.12 Bq L^−1^. Inner 5 mm has a lower 4 h RMSE only in S23 and H29; Old has a lower RMSE in the other 17 compositions. The reduction is only 0.6% in S23 but is substantial in H29. Reduced sensitivity to interference therefore does not imply a lower total error in every composition.

In H29, at a tritium concentration of 100 Bq L^−1^, the 600 s RMSE is 81.8 Bq L^−1^ for Old, 84.6 for Inner 5 mm and 102.1 for Inner 10 mm. At this acquisition time, the reduction in bias does not offset the increase in counting uncertainty. At 3600 s, Inner 5 mm reduces RMSE from 45.4 to 36.8 Bq L^−1^, or by 19.1%. At 14,400 s, RMSE decreases from 37.02 to 21.95 Bq L^−1^, a reduction of 40.7%; the corresponding reduction at the zero tritium concentration is 44.8%. For this stationary composition at 100 Bq L^−1^, the RMSE curves for Old and Inner 5 mm intersect at approximately 888 s. This behavior reflects the combination of persistent bias and counting variance that decreases as 1/t.

**Figure 4:**
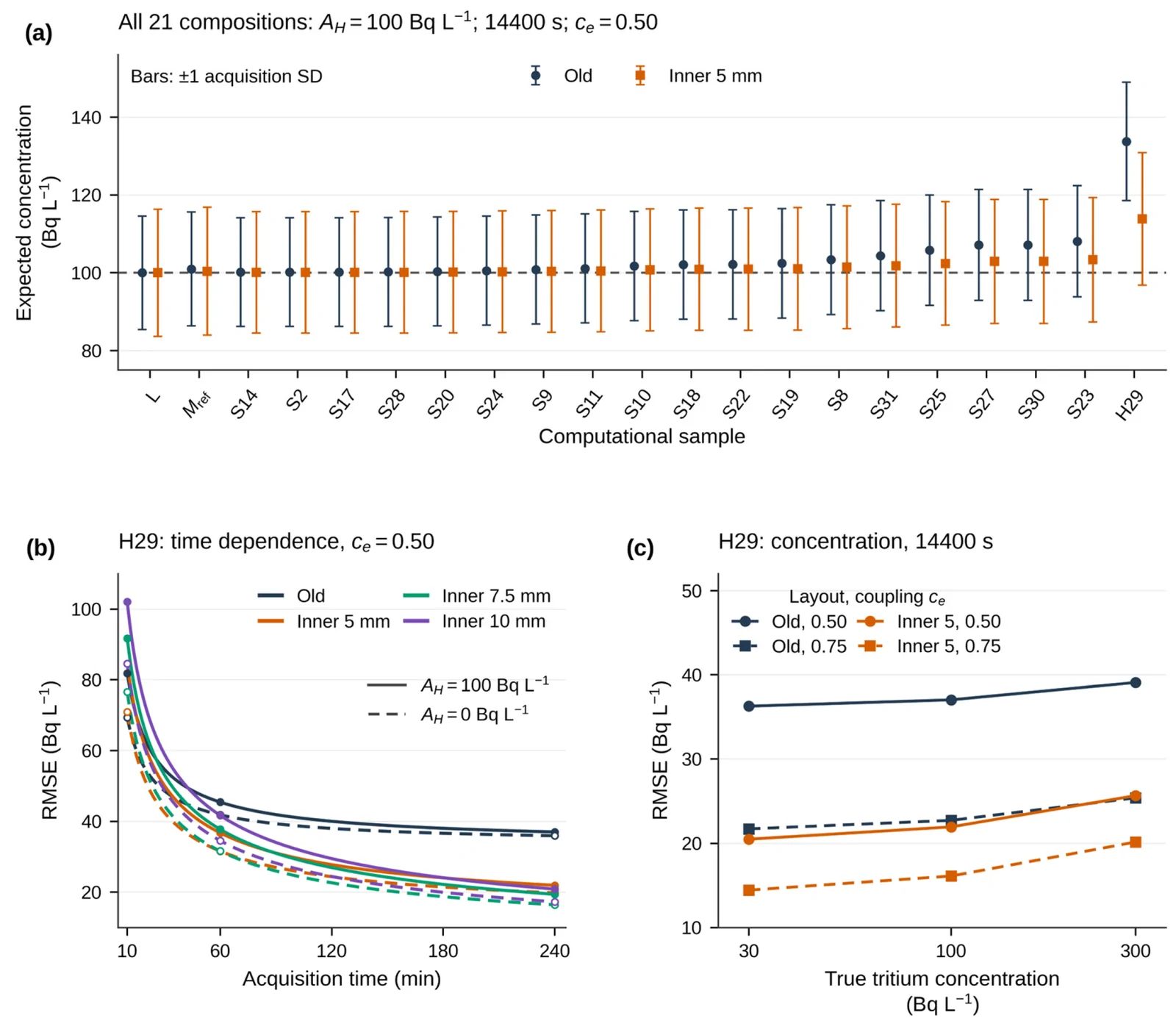
Predicted mean tritium concentrations and counting uncertainty across the modeled seawater compositions. (**a**) Model-predicted mean concentrations for Old and Inner 5 mm in L, M_ref_ and the 19 literature-based station-pair compositions, at a true tritium concentration of 100 Bq L^−1^, c_e_ = 0.50 and 14,400 s. The horizontal dashed line marks the true concentration. Error bars show ±1 standard deviation for a single acquisition; they are not confidence intervals for the mean or model parameters. (**b**) H29 RMSE for Old, Inner 5 mm, Inner 7.5 mm and Inner 10 mm from 600 to 14,400 s at c_e_ = 0.50. Solid and dashed lines indicate true tritium concentrations of 100 and 0 Bq L^−1^, respectively. The curves use variance scaling for a stationary response; markers identify the calculated values at 600, 3600 and 14,400 s. (**c**) H29 RMSE for Old and Inner 5 mm at 14,400 s and true concentrations of 30, 100 and 300 Bq L^−1^, comparing c_e_ = 0.50 and 0.75. The concentration axis is logarithmic, and connecting lines guide the eye between the specified test concentrations. All panels retain each layout’s L calibration and fixed readout cuts.

Figure 4 compares performance across the full set of compositions and shows its time dependence in H29. Most station pairs produce much smaller transfer errors than H29; extending acquisition to 4 h therefore need not compensate for the loss of sensing response. In the separate near-calibration composition M_ref_, the biases are 0.97 Bq L^−1^ for Old and 0.40 for Inner 5 mm, but Old retains a lower 4 h RMSE. Within the tested set, the dual-diameter geometry provides its greatest measurement benefit when composition changes cause appreciable bias under the retained calibration.

Varying the tritium concentration while holding the interference composition fixed provides a further test of modeled performance (Table 6). In H29, at 4 h and c_e_ = 0.50, true concentrations of 30, 100 and 300 Bq L^−1^ yield predicted mean concentrations of 43.85, 113.85 and 313.85 Bq L^−1^ for Inner 5 mm, compared with 63.75, 133.75 and 333.75 Bq L^−1^ for Old. Inner 5 mm reduces RMSE by 43.5%, 40.7% and 34.3%, respectively. The additive interference bias is independent of tritium concentration, whereas the signal counting variance increases with concentration. Consequently, RMSE relative to the true concentration decreases as concentration increases, even though absolute RMSE rises. The H29 benefit therefore persists across the three prescribed tritium concentrations.

### 3.2. Interference Rejection and Background Reference Response

**Figure 5:**
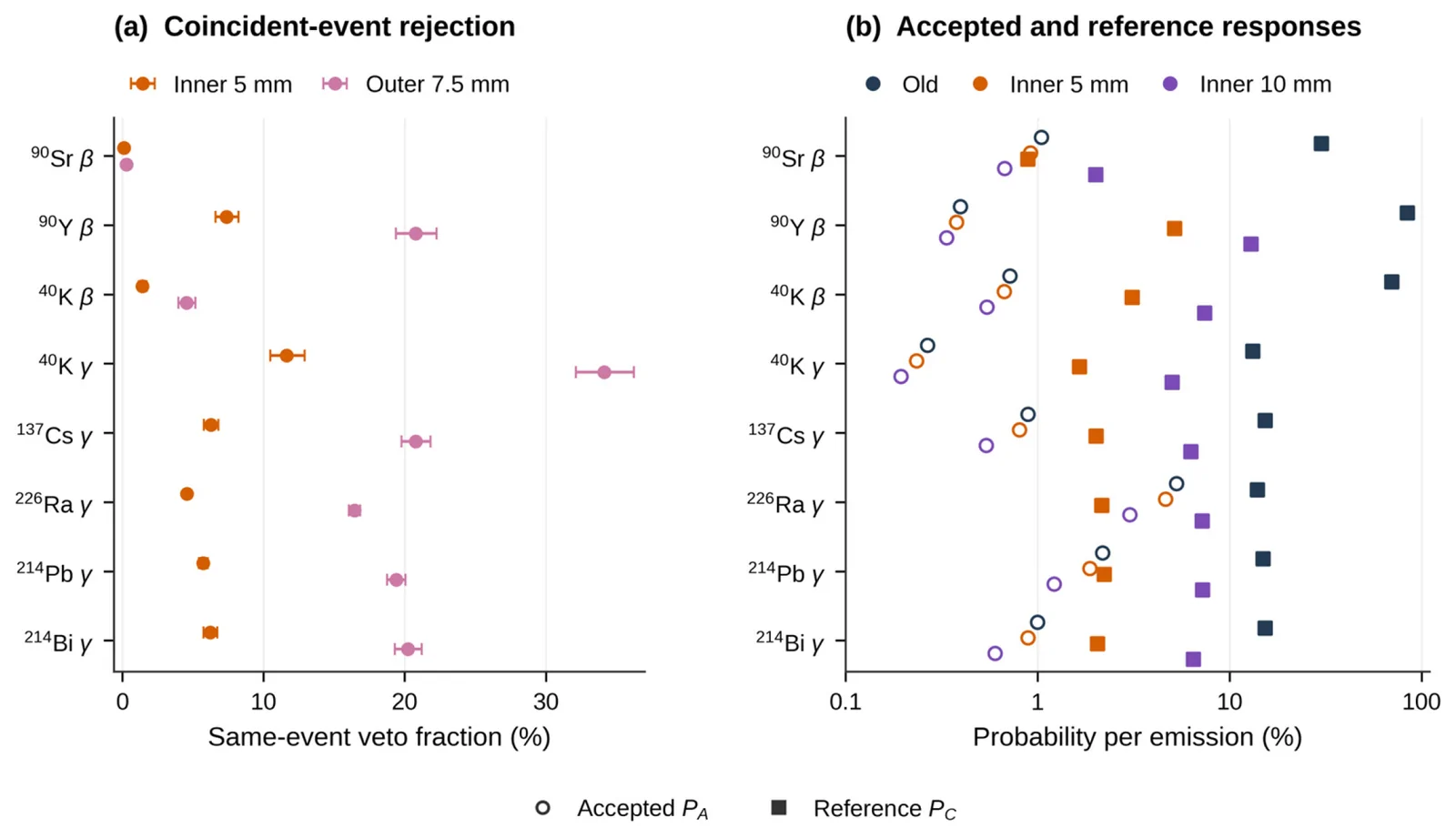
Direct deposited-energy transport responses for the eight modeled background emissions. (**a**) Fraction of small-fiber tritium-window candidates rejected by a coincident large-fiber energy deposit of at least 18.6 keV for Inner 5 mm and Outer 7.5 mm. Points show (n_ROI_ − n_after_)/n_ROI_, with two-sided 95% Wilson score intervals calculated using n_ROI_ as the binomial denominator. (**b**) Accepted-channel and reference-channel probabilities per emitted primary for Old, Inner 5 mm and Inner 10 mm; open circles denote P_A_ and filled squares denote P_C_. Each source–layout simulation contains n = 10^6^ primary histories. For Old, A is defined by 0 < E_s_ ≤ 18.6 keV and C by E_s_ > 18.6 keV; for the dual-diameter layouts, A additionally requires E_l_ < 18.6 keV, and C is defined by E_l_ ≥ 18.6 keV. E_s_ and E_l_ are the event-level energies deposited in the small- and large-fiber channels, respectively. Panel (**b**) uses a logarithmic probability axis and reports point estimates without weighting by activity concentration or emission yield. These results are obtained directly from transport tallies, without photoelectron reconstruction. The tritium signal is excluded from these background panels.

Table 7 reports channel probabilities normalized per emission. For Inner 5 mm, the reference-channel probability for ^40^K β is 3.1019%, compared with 0.6701% in the accepted channel, yet the same-event veto removes only 1.41% of the raw ^40^K β candidates. Outer 7.5 mm rejects 34.13% of ^40^K γ candidates and 20.78% of ^137^Cs γ candidates, but only 4.53% of ^40^K β candidates. The independent reference channel provides background information even when a tritium-like deposit does not produce a simultaneous large-fiber trigger (Figure 5). The measurement benefit therefore depends on statistical background tracking as well as event rejection.

Reference counts alone do not determine the quality of the correction. In L at 600 s, the deposited-energy model predicts 1051.68 reference counts and 11.03 accepted background counts for Old, compared with 48.07 and 10.17 for Inner 5 mm. Old therefore has lower relative counting uncertainty in the reference channel. Correction quality also depends on how consistently the reference response predicts the accepted background as the relative radionuclide contributions change.

**Figure 6:**
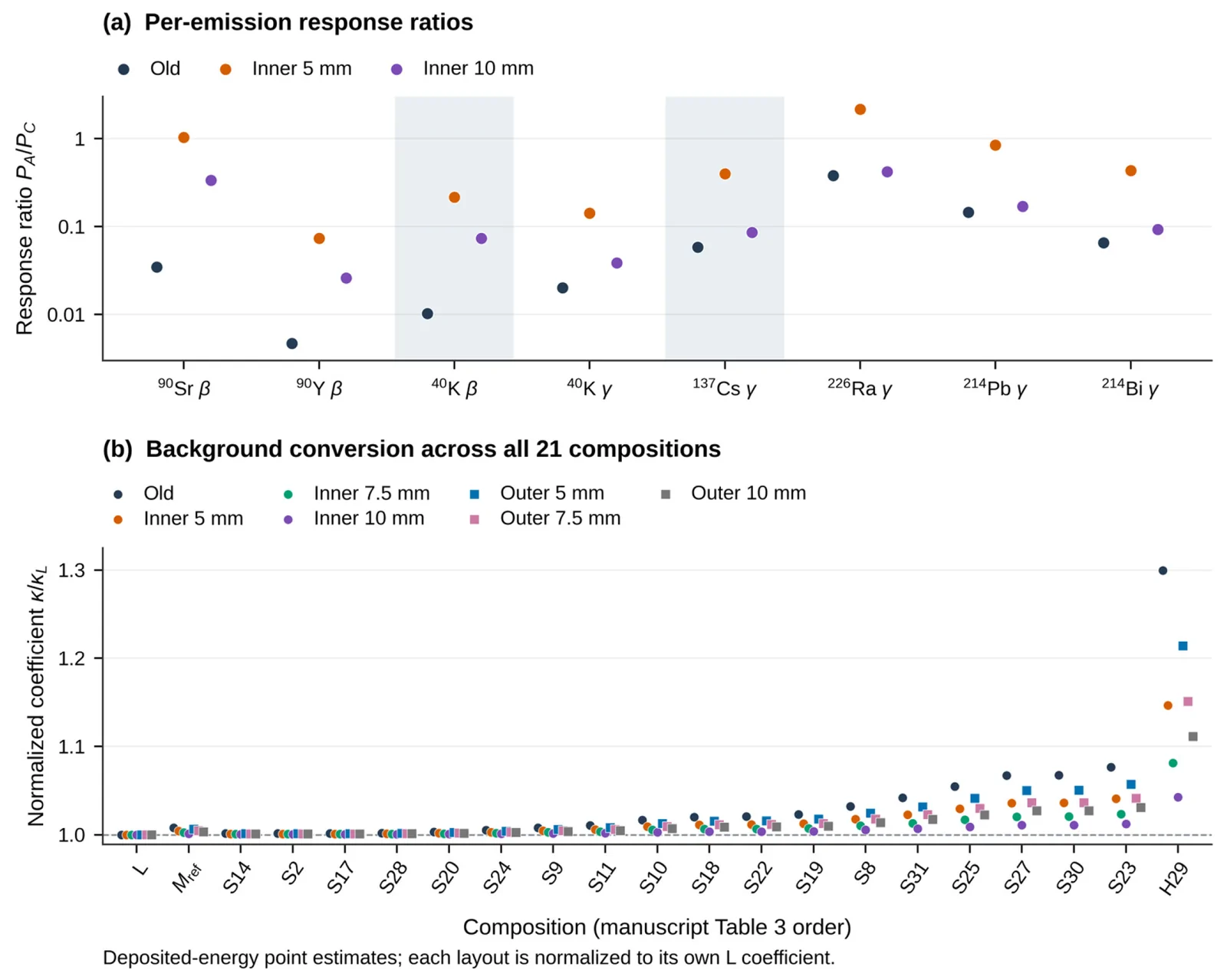
Deposited-energy response ratios and composition dependence of the background conversion coefficient. (**a**) P_A_/P_C_ for the eight modeled background emissions in Old, Inner 5 mm and Inner 10 mm, shown on a logarithmic axis. Shading identifies the ^40^K β and ^137^Cs γ components discussed in the text. (**b**) Background conversion coefficient κ normalized to each layout’s L value for all seven layouts and all 21 compositions, ordered as in Table 3: L, M_ref_ and the 19 station-based compositions ending with H29. Symbols are offset horizontally for readability. Each position represents a separate composition; no interpolation between samples is implied. The dashed line marks κ/κ_L_ = 1. For each composition, κ is calculated by weighting the background responses by activity concentration and emission yield, with tritium excluded. Probabilities are derived directly from n = 10^6^ primary transport histories per source–layout combination using the deposited-energy channel definitions in Figure 5. Ratios and normalized coefficients are point estimates; transport uncertainty has not been propagated to these plotted quantities. This figure does not use photoelectron reconstruction.

The accepted-to-reference ratios explain the differences in composition sensitivity. For Old, P_A_/P_C_ is 0.01026 for ^40^K β and 0.05819 for the selected ^137^Cs γ line; the corresponding values for Inner 10 mm are 0.07346 and 0.08593. More similar ratios reduce sensitivity to changes in the relative contributions of these emissions. From L to H29, the deposited-energy coefficient κ changes by 30.0% for Old, 14.7% for Inner 5 mm and 4.3% for Inner 10 mm (Figure 6). With the L calibration retained, the H29 biases are 21.3, 12.2 and 4.2 Bq L^−1^, respectively. These direct transport results support the interference mechanism identified in the PE calculation.

Holding the natural matrix fixed across the 19 station-based compositions isolates the effects of variation in the Sr/Cs pair. Equation (10) weights each change in activity concentration by the corresponding tritium-equivalent response. Proportional increases in all background components leave κ unchanged; changes in their relative contributions cause the calibration-transfer error examined here. In L, ^40^K β dominates the accepted interference. Its small same-event veto fraction explains why statistical background tracking remains essential even when gamma-event rejection is effective.

Reduced bias in H29 is accompanied by lower false-positive probabilities. At c_e_ = 0.50, the actual probabilities at 600 s are 12.3%, 5.7% and 3.5% for Old, Inner 5 mm and Inner 10 mm, respectively; at 4 h, they are 88.2%, 24.7% and 4.1%. Table 8 identifies the station-based compositions that satisfy the 5% criterion at 4 h, with zero tritium and each layout’s L coefficient retained. Old meets the criterion in S14, S2, S17, S28 and S20. Inner 5 mm meets it in those five compositions and in S24, S9 and S11; Inner 10 mm meets it in all 19. L and M_ref_ are excluded from these totals. These finite-count results apply to the specified optical setting, acquisition time and backgrounds. When composition-correct coefficients are used, all evaluated conditional tests satisfy the nominal bound under the assumptions in Section 2.5. Lower measurement RMSE and control of false positives remain distinct criteria.

Using the response probabilities and background moments of the same model, the conditional-binomial rule was also evaluated for H29 with each layout’s L coefficient fixed at c_e_ = 0.50. The calculation follows Section 2.5, including tritium leakage into C and an omitted Poisson-tail probability below 10^−13^.

At 100 Bq L^−1^ tritium, the detection probabilities are 63.37% for Old and 40.23% for Inner 5 mm at 600 s and exceed 99.999% for both at 4 h. Under the same rule with zero tritium, the respective false-positive probabilities are 12.30% and 5.75% at 600 s, and 88.22% and 24.73% at 4 h. Thus, reduced transfer bias lowers false-positive frequency, but the high 4 h detection probability does not imply 5% false-positive control under the H29/L mismatch.

### 3.3. Retained Tritium Response and MDA

Reference fibers displace part of the fine sensing array. Inner 5 mm retains 87.32% of Old’s small-fiber lateral area and 88.25% of its deposited-energy concentration sensitivity G. Inner 10 mm retains 57.06% of G, and Outer 7.5 mm retains 43.53%. Old has a tritium response probability of 0.1021%. Figure 2 relates these full-array responses to the single-fiber trends and the associated geometric changes. The design objective is to limit signal loss while obtaining a reference response that is less sensitive to changes in interference composition.

At baseline coupling, the mid-fiber photoelectron yield is 0.1700 primary PE keV^−1^ per end. A 5.7 keV deposit produces a mean total of 1.94 PE and a two-ended trigger probability of approximately 0.385. Integrating over the deposited-energy distribution gives tritium acceptance probabilities of 0.03715%, 0.03332% and 0.02422% for Old, Inner 5 mm and Inner 10 mm, respectively (Table 4). After reference-channel leakage is included, their effective concentration sensitivities have a ratio of 1:0.8881:0.5763. The 5 mm cluster therefore preserves substantially more tritium response than the larger-diameter alternative.

Inner 5 mm has the lowest composition-calibrated MDA among the six dual-diameter candidates. At 600 s, its deposited-energy MDA is 120.5 Bq L^−1^ in L and 131.8 Bq L^−1^ in H29 (Table 9). The corresponding values are 152.9 and 161.7 Bq L^−1^ for Inner 10 mm and 97.7 and 105.9 Bq L^−1^ for Old. The 5 mm central cluster therefore provides the best counting sensitivity within the tested dual-diameter family. Its lower interference-induced bias is a separate measurement advantage over the conventional array.

The PE calculation preserves this ordering of MDA values (Table 4). At 600 s, Inner 5 mm has an MDA of 296.6 Bq L^−1^ in L and 314.3 Bq L^−1^ in H29, compared with 248.3 and 261.9 Bq L^−1^ for Old. This represents an increase of approximately 20%, while 88.8% of the effective response is retained. At 4 h, the corresponding values are 47.3 and 50.6 Bq L^−1^ for Inner 5 mm, compared with 40.7 and 43.3 Bq L^−1^ for Old. The calculation includes photon fluctuations, two-ended selection and reference-channel leakage, with the correct background coefficient applied to each composition. These values quantify composition-calibrated counting sensitivity; they do not represent detection limits when a change in interference remains uncorrected.

**Figure 7:**
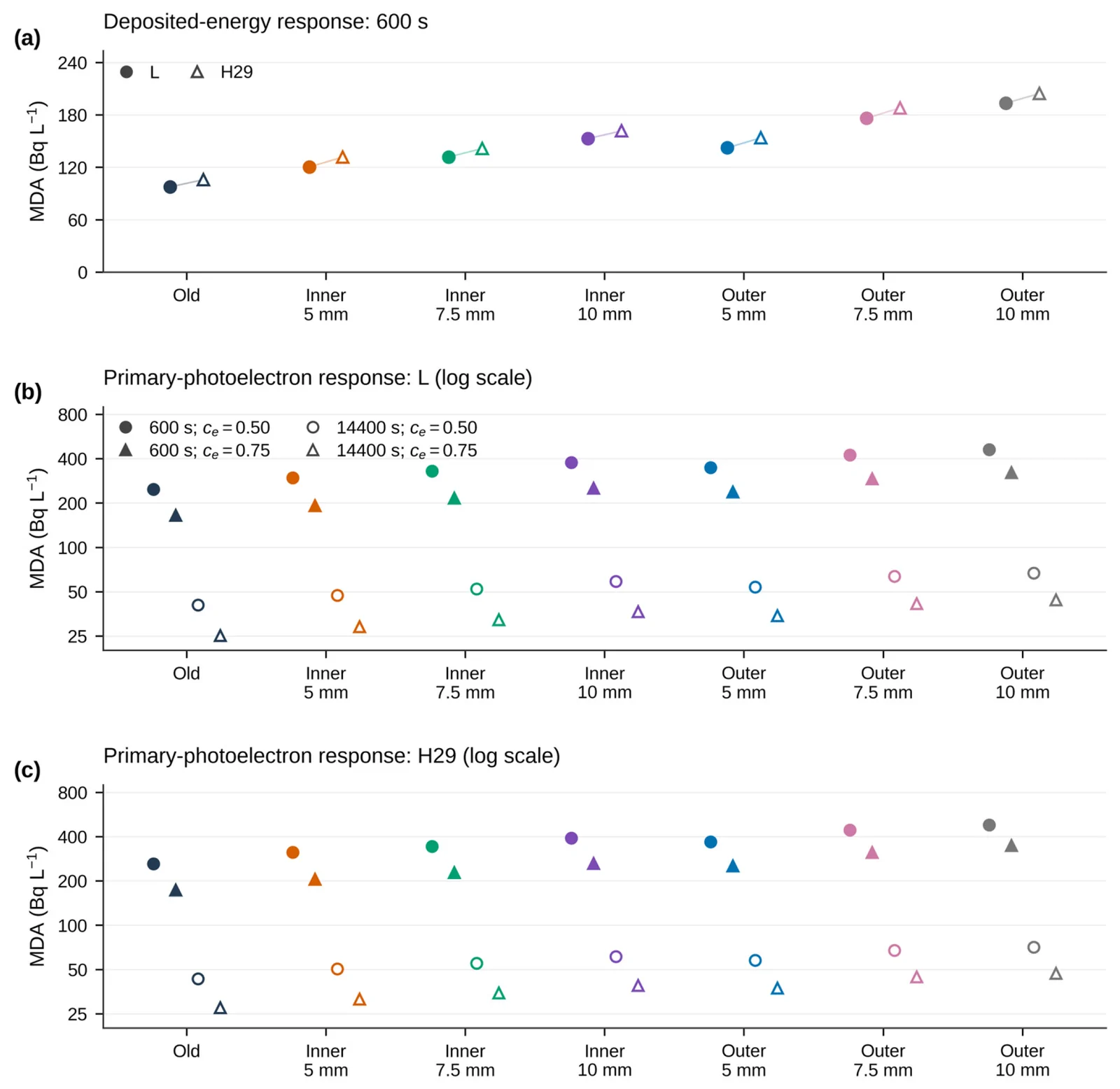
Minimum detectable activity concentration with the background composition correctly specified. (**a**) Deposited-energy MDAs for all seven layouts in L and H29 at 600 s; each linked pair represents one layout. (**b**,**c**) Primary-photoelectron MDAs in L and H29, respectively, shown on logarithmic vertical axes. Filled and open markers denote 600 and 14,400 s, respectively; circles and triangles denote common coupling values of c_e_ = 0.50 and 0.75. Layout colors are consistent across panels, and readout cuts remain fixed. Each MDA uses the corresponding composition-specific background coefficient, the exact conditional-binomial test with a nominal one-sided α = 0.05, and a target detection power of 0.95. Coupling comparisons assess model sensitivity and are not confidence intervals. These composition-calibrated sensitivity benchmarks use a different calibration premise from the predicted mean concentrations obtained with the fixed L calibration in Figure 4.

With the PE cuts held fixed, increasing the common coupling, c_e_, from 0.50 to 0.75 reduces the 600 s MDA in L from 248.3 to 164.8 Bq L^−1^ for Old, from 296.6 to 192.4 for Inner 5 mm, and from 377.8 to 252.2 for Inner 10 mm. In a separate test, an assumed accepted-channel residual rate of 0.01 s^−1^, with r_C_ = 0 and recalibrated coefficients, raises the baseline MDA in L to 304.0, 389.2 and 508.3 Bq L^−1^, respectively. This residual rate is an input to the sensitivity analysis, not a measured instrumental background. Figure 7 compares MDA under the common readout conditions.

### 3.4. Readout Sensitivity and Design Selection

**Figure 8:**
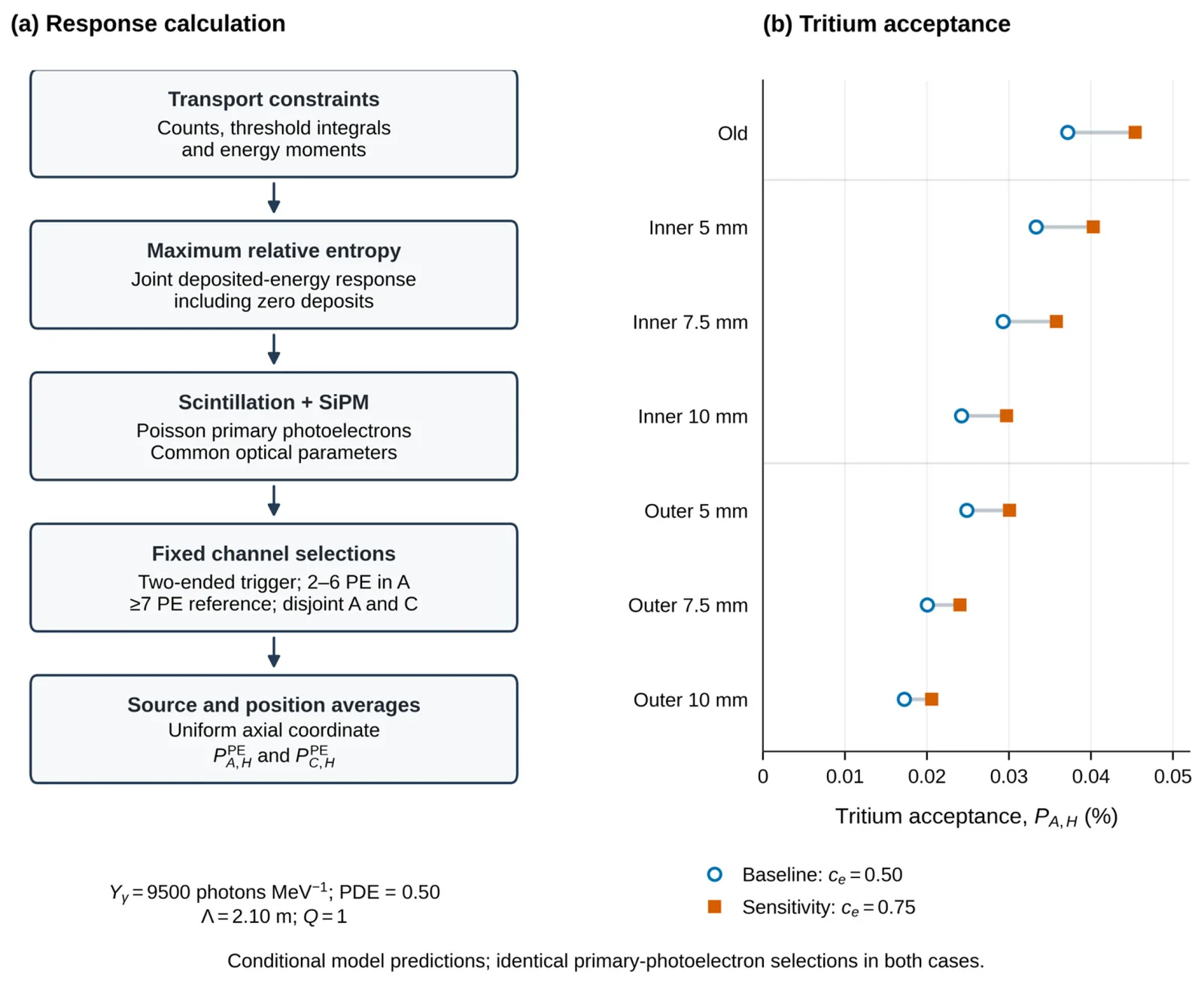
Optical-response calculation and source-integrated tritium acceptance. (**a**) Calculation workflow linking transport constraints, maximum-relative-entropy reconstruction of the deposited-energy response, scintillation and SiPM conversion, fixed primary-photoelectron selections, and averaging over the source response and axial position. (**b**) Source-integrated tritium acceptance probability, P_A,H_, for all seven layouts at effective coupling c_e_ = 0.50 (baseline) and 0.75 (sensitivity calculation). The common parameters are Yγ = 9500 photons MeV^−1^, PDE = 0.50, attenuation length Λ = 2.10 m and relative light yield Q = 1. Each of the two channel ends must register at least one primary photoelectron. The small-channel acceptance window is 2–6 PE; the reference threshold is 7 PE in the small-fiber channel for Old and in the large-fiber channel for the dual-diameter layouts. Accepted and reference events are disjoint. All cuts remain fixed across coupling settings. Gray segments connect the two conditional predictions for each layout and do not represent uncertainty. The probabilities include zero-deposit histories and averaging over a uniform axial-position distribution. These are conditional model predictions, without additional transport simulations or measured optical spectra.

All 189 source–geometry reconstructions reproduce the transport probability constraints within 4.36 × 10^−8^. Grid refinement changes representative MDA values in L by less than 0.2%. Replacing the uniform energy measure with a log-energy measure gives MDA values in L of 257.9, 310.4 and 384.5 Bq L^−1^ for Old, Inner 5 mm and Inner 10 mm, respectively, compared with baseline values of 248.3, 296.6 and 377.8 Bq L^−1^. These checks distinguish discretization error from sensitivity to the assumed energy distribution. Figure 8 shows the transport and readout stages and the common optical settings.

The predicted concentration bias is more sensitive to energy reconstruction than the calibrated MDA. In H29, the baseline PE model overestimates the concentration by 33.7 Bq L^−1^ for Old and 13.8 Bq L^−1^ for Inner 5 mm, but underestimates it by 1.0 Bq L^−1^ for Inner 10 mm. With log-energy reconstruction, Old overestimates the concentration by 20.2 Bq L^−1^, whereas Inner 5 mm and Inner 10 mm underestimate it by 1.6 and 15.3 Bq L^−1^, respectively. The near-zero baseline bias of Inner 10 mm therefore depends on cancellation among source contributions. At 4 h and 100 Bq L^−1^, the log-energy model gives H29 RMSE values of 25.36 Bq L^−1^ for Old and 17.25 Bq L^−1^ for Inner 5 mm, preserving a reduction of 32.0%. Among the three Inner layouts evaluated under this reconstruction, Inner 5 mm has the lowest mean RMSE across the 19 compositions, although Old retains the lower ensemble mean.

Increasing the common coupling to c_e_ = 0.75 lowers MDA while the selection cuts remain fixed (Table 10). Inner 5 mm retains 87.8% of Old’s effective response and has composition-calibrated 4 h MDAs of 28.9 Bq L^−1^ in L and 31.5 Bq L^−1^ in H29. Its absolute bias in H29 is 10.5 Bq L^−1^, compared with 19.8 Bq L^−1^ for Old. At a tritium concentration of 100 Bq L^−1^ and an acquisition time of 4 h, its RMSE is 16.12 Bq L^−1^, compared with 22.72 Bq L^−1^ for Old, a reduction of 29.1%. Across the tests at 30, 100 and 300 Bq L^−1^, the reductions are 33.5%, 29.1% and 20.6%, respectively. Improved collection therefore lowers measurement RMSE while the reference cluster continues to reduce interference bias.

Among the six dual-diameter layouts, a 5 mm central reference cluster with c_e_ = 0.75 is the preferred candidate when both low ensemble-mean RMSE and retained counting sensitivity are considered. At 100 Bq L^−1^ and 4 h, its mean RMSE across the 19 compositions is 11.72 Bq L^−1^, compared with 12.69 and 14.21 Bq L^−1^ for Inner 7.5 mm and Inner 10 mm, respectively; the three Outer layouts have mean RMSE values of 14.18, 16.30 and 17.39 Bq L^−1^. Inner 5 mm also has the lowest composition-calibrated MDA among the dual-diameter layouts in L and H29. Old’s ensemble mean remains slightly lower, at 11.27 Bq L^−1^. If the sole objective is to minimize H29 RMSE, Inner 7.5 mm gives 14.66 Bq L^−1^, below the 16.12 Bq L^−1^ for Inner 5 mm, but its calibrated MDA increases from 31.5 to 34.6 Bq L^−1^. These comparisons use identical optical settings and acquisition times. Choosing the 5 mm cluster does not establish 5% false-positive control for arbitrary compositions under fixed calibration. Composition-calibrated MDA and the actual false-positive probabilities in Section 3.2 refer to different calibration conditions. Changes in coupling and energy reconstruction are evaluated as separate sensitivity tests.

### 3.5. Representative Direct-Event Check

A representative numerical check evaluates the tritium response of Old and Inner 5 mm directly from event-level sensing and reference energies. For each geometry, 10^6^ histories with the original source settings and seed reproduce the aggregate transport tallies, while a further 5 × 10^6^ histories use an independent seed. Zero-deposit histories are retained. The recorded energies are integrated with the same primary-photoelectron selection, common uniform axial-position kernel and c_e_ = 0.50, using 32-point axial quadrature.

The original-seed direct P_A,H_ values are 0.03728% for Old and 0.03344% for Inner 5 mm, compared with reconstructed values of 0.03715% and 0.03332%; the relative differences are +0.34% and +0.36%. Independent-seed estimates are 0.03822% and 0.03387%, with Monte Carlo standard errors of 0.00060 and 0.00057 percentage points, respectively. These errors describe only the direct estimates. The original-seed comparison is an internal check because it shares the histories used to constrain the reconstruction.

This comparison checks the integrated tritium acceptance for two representative layouts. It does not establish agreement of the complete joint spectra or validate the mixed-background response; broader spectral benchmarking remains a useful extension of the present model.

## 4. Discussion

### 4.1. Structural Basis of Improved Tritium Measurement

The structural benefit arises from a closer correspondence between the reference response and the interference entering the tritium window. An energetic electron can deposit a small amount of energy in a fine sensing fiber without triggering a coincident veto; the independent reference channel nevertheless provides a statistical estimate of the remaining interference. Performance therefore depends on both event rejection and the stability of the accepted-to-reference ratio. This explains why modest same-event beta rejection can accompany substantial bias reduction. The conventional array also uses simultaneous reference counts, so the comparison assesses how geometry affects the reference response.

### 4.2. Interference Tolerance and Retained Sensitivity

The 5 mm central cluster retains approximately 89% of the effective tritium response while reducing sensitivity to composition changes. Under baseline collection conditions, its 600 s calibrated MDA is approximately 20% higher than Old’s, but the largest 4 h RMSE across the 19 station-based compositions at 100 Bq L^−1^ decreases from 37.02 to 21.95 Bq L^−1^. The ensemble mean increases by 2.6%, from 15.72 to 16.12 Bq L^−1^. Thus, a modest increase in mean RMSE accompanies a substantially smaller error in the composition with the greatest transfer bias. Equal weighting describes the tested set and does not represent environmental occurrence frequencies. Under the modeled conditions, this geometry may be useful when tolerance of appreciable interference changes is required alongside counting sensitivity, but it offers little measurement benefit when the calibration remains representative.

The intended application is seawater tritium measurement under variable radionuclide interference. The computational matrix represents seawater and the specified dissolved radionuclides, but does not cover the full range of water chemistry or optical turbidity. Absolute predictions remain conditional on the simplified emission library, constrained energy-response reconstruction and radiation-only primary-PE model. Prototype validation of the low-energy response, instrumental background and calibration transfer is needed to establish operational performance. The prescribed concentrations of 30, 100 and 300 Bq L^−1^ test modeled measurement performance; they do not establish the detector’s application range. Concentration results must be interpreted with their MDA, counting time and calibration conditions. Four-hour integration improves counting precision when composition and detector response remain approximately stationary. The acquisition interval should therefore reflect the acceptable concentration error and required update rate.

### 4.3. Prototype Development and Future Work

Building on the team’s previous fiber-detector work [16,17,18], a preliminary design for the Inner 5 mm configuration has been completed. Figure 3 presents this structural design only; the physical detector is still under development. The design includes a nominal 50 mm inner-diameter water chamber containing 1122 sensing fibers of 1 mm diameter and seven central reference fibers of 5 mm diameter. At each end, four sensing-sector routes and seven reference routes feed optically separated dry heads. Signals are grouped into the sensing and reference end signals used in the model. The active water volume remains free of sector partitions.

The present paper establishes the structural and estimation concept through simulation. Experimental work will assess position-dependent light collection, readout noise and timing, followed by blanks and samples of independently determined tritium activity under varying interference. Full decay-source treatment, broader spectral benchmarking and combined response/calibration uncertainty will support this development. These extensions will test how the predicted reduction in interference bias transfers to instrument operation; the design drawing itself represents engineering progress, not measured detector performance.

## 5. Conclusions

This simulation study uses dual-diameter scintillating fibers and a separately read out reference channel to reduce bias in estimated tritium activity concentration caused by changes in complex seawater backgrounds. Within the specified simulation model, Inner 5 mm reduces concentration bias across all 19 literature-based Sr/Cs compositions. In the case with the greatest interference, it lowers 4 h RMSE by 40.7% at 100 Bq L^−1^ and by 34.3–43.5% across the 30–300 Bq L^−1^ tests. It retains 88.8% of the baseline effective response and has the lowest calibrated MDA among the new layouts. At a common coupling of 0.75, its 4 h MDAs are 28.9 Bq L^−1^ in L and 31.5 Bq L^−1^ in H29, and its ensemble-mean RMSE is the lowest among the tested dual-diameter layouts. At this coupling, the 7.5 mm central cluster minimizes H29 RMSE at 100 Bq L^−1^ and 4 h, with a higher calibrated MDA. Old retains a lower calibrated MDA and better precision for small composition changes. Geometry selection therefore depends on the measurement objective, with measurement error, counting sensitivity and false-positive control assessed under their respective calibration conditions. Prototype development and experimental characterization form the next stage of this work.

## Figures and Tables

**Table 1:** Geometry and tritium response of the tested arrays.

Layout	Large D (mm)	Small Fibers	Large Fibers	Water Volume (L)	Sensing Surface (m^2^)	ε_H_ (%)
Old	—	1285	0	0.23858	1.0092	0.1021
Inner 5	5	1122	7	0.23623	0.8811	0.0910
Inner 7.5	7.5	972	7	0.22273	0.7633	0.0809
Inner 10	10	726	7	0.21090	0.5702	0.0659
Outer 5	5	769	26	0.21228	0.6039	0.0670
Outer 7.5	7.5	583	16	0.19971	0.4579	0.0531
Outer 10	10	421	11	0.19225	0.3306	0.0454

The small fibers are 1 mm in diameter; the chamber and all fibers are 250 mm long. The reported areas are lateral surface areas. ε_H_ denotes the deposited-energy acceptance probability per tritium decay.

**Table 2:** Radiation-emission inputs for the transport model.

Emission	Energy Type	Energy (keV)	Yield b_i_ (per Decay)	Daughter Z
^3^H β	β endpoint	18.6	1.0000	2
^90^Sr β	β endpoint	546	1.0000	39
^90^Y β	β endpoint	2280	1.0000	40
^40^K β	β endpoint	1310	0.8928	20
^40^K γ	γ line	1460.82	0.1066	—
^137^Cs γ	γ line	661.657	0.8510	—
^226^Ra γ	γ line	186.211	0.0359	—
^214^Pb γ	γ line	351.932	0.3580	—
^214^Bi γ	γ line	609.312	0.4610	—

Each emission yield is applied once to the corresponding parent activity concentration. The input library contains the listed discrete γ lines and approximate β spectra; it does not represent complete decay schemes.

**Table 3:** Literature-based computational sample compositions for the structural comparison (Bq L^−1^).

Sample	Sr/Cs Date	^40^K	^226^Ra	^90^Sr	^137^Cs
L	—	11.51	0.00275	0.00074	0.00146
M_ref_	26 August 2011	11.55	0.00336	0.0037	0.1
S14	11 June 2011	9.99	0.0093	0.0011	0.00226
S2	7 June 2011	9.99	0.0093	0.0014	0.00258
S17	12 June 2011	9.99	0.0093	0.0011	0.00328
S28	16 June 2011	9.99	0.0093	0.0011	0.0079
S20	13 June 2011	9.99	0.0093	0.0017	0.0221
S24	14 June 2011	9.99	0.0093	0.0025	0.043
S9	9 June 2011	9.99	0.0093	0.0032	0.0712
S11	10 June 2011	9.99	0.0093	0.0078	0.0991
S10	10 June 2011	9.99	0.0093	0.0093	0.166
S18	12 June 2011	9.99	0.0093	0.0061	0.203
S22	13 June 2011	9.99	0.0093	0.0069	0.21
S19	12 June 2011	9.99	0.0093	0.0088	0.235
S8	9 June 2011	9.99	0.0093	0.0056	0.335
S31	17 June 2011	9.99	0.0093	0.015	0.438
S25	15 June 2011	9.99	0.0093	0.017	0.58
S27	15 June 2011	9.99	0.0093	0.033	0.713
S30	17 June 2011	9.99	0.0093	0.028	0.717
S23	14 June 2011	9.99	0.0093	0.026	0.818
H29	16 June 2011	9.99	0.0093	0.085	3.46

L is the Ningde calibration composition [19]. M_ref_ combines the Fuqing natural matrix with the station-20 Sr/Cs pair collected on 26 August 2011 [19,24]. S labels identify KOK 2011 stations; H29 denotes station 29, whose matched pair has the highest Cs activity concentration. All 19 uniquely matched pairs are retained and ordered by increasing sampling-date-corrected Cs concentration [23,25,26]. Their K/Ra inputs are independent Yantai regional means [20], not measurements from the KOK stations. Dates identify the Sr/Cs samples. The relations ^90^Y = ^90^Sr and ^214^Pb = ^214^Bi = ^226^Ra are model assumptions. These are computational compositions, not new field measurements of complete radionuclide composition.

**Table 4:** Source-integrated primary-photoelectron response and composition-calibrated MDAs at 600 s and 4 h.

Layout	^3^H P_A_ (%)	G/G_Old_ in L	L MDA 600 s	H29 MDA 600 s	L MDA 4 h	H29 MDA 4 h
Old	0.0371	1.0000	248.3	261.9	40.7	43.3
Inner 5	0.0333	0.8881	296.6	314.3	47.3	50.6
Inner 7.5	0.0293	0.7360	329.8	344.4	52.3	55.2
Inner 10	0.0242	0.5763	377.8	391.1	58.7	61.4
Outer 5	0.0248	0.5952	349.1	369.1	53.9	57.7
Outer 7.5	0.0200	0.4513	424.2	443.6	63.6	67.6
Outer 10	0.0172	0.3735	461.2	482.1	67.0	71.0

MDA units: Bq L^−1^. Nominal c_e_ = 0.50 and mean PDE = 0.50; radiation-only baseline. The reconstructed deposited-energy distribution is convolved with Poisson primary-photoelectron statistics and averaged over axial position. Each MDA uses its composition-specific correct background coefficient. G includes tritium leakage into the reference channel.

**Table 5:** Predicted mean tritium concentrations and 4 h measurement errors with the L calibration retained for all compositions.

Sample	Old E[Â_H_]	Inner 5 E[Â_H_]	Old RMSE 4 h	Inner 5 RMSE 4 h
L	100.00	100.00	14.59	16.35
M_ref_	100.97	100.40	14.67	16.41
S14	100.15	100.10	13.97	15.61
S2	100.16	100.10	13.97	15.61
S17	100.16	100.10	13.97	15.61
S28	100.21	100.12	13.97	15.62
S20	100.35	100.18	13.98	15.62
S24	100.55	100.26	14.00	15.63
S9	100.82	100.37	14.02	15.65
S11	101.11	100.48	14.05	15.67
S10	101.76	100.75	14.14	15.71
S18	102.10	100.90	14.20	15.72
S22	102.17	100.92	14.22	15.73
S19	102.42	101.02	14.27	15.75
S8	103.37	101.42	14.49	15.82
S31	104.40	101.83	14.80	15.91
S25	105.77	102.40	15.32	16.04
S27	107.12	102.92	15.93	16.20
S30	107.14	102.94	15.93	16.19
S23	108.10	103.34	16.42	16.31
H29	133.75	113.85	37.02	21.95

True tritium concentration: 100 Bq L^−1^; expected measurement results and errors are in Bq L^−1^. Values below or above 100 indicate underestimation or overestimation. The baseline PE model uses c_e_ = 0.50, fixed L calibration and 14,400 s. Entries are analytical model predictions. L checks calibration consistency; M_ref_ is an external small-perturbation composition. The 19 station compositions are reported without a performance filter. RMSE combines bias and counting variance.

**Table 6:** Predicted tritium measurement performance at different true concentrations in H29 at 4 h.

c_e_	True ^3^H (Bq L^−1^)	Old E[Â_H_]	Inner 5 E[Â_H_]	Old RMSE	Inner 5 RMSE
0.50	30	63.75	43.85	36.27	20.49
0.50	100	133.75	113.85	37.02	21.95
0.50	300	333.75	313.85	39.08	25.66
0.75	30	49.82	40.54	21.71	14.44
0.75	100	119.82	110.54	22.72	16.12
0.75	300	319.82	310.54	25.39	20.15

Concentrations and errors are in Bq L^−1^. The background composition H29, PE cuts and 14,400 s acquisition are fixed. Each coupling setting retains its corresponding L calibration. Only the specified true tritium concentration changes within each coupling series. These are model-based concentration estimates, not experimental sample measurements.

**Table 7:** Accepted-channel and reference-channel probabilities per emission (%).

Emission	Old P_A_	Old P_C_	Inner 5 P_A_	Inner 5 P_C_	Inner 10 P_A_	Inner 10 P_C_
^3^H β	0.1021	0.0000	0.0910	0.0000	0.0659	0.0000
^90^Sr β	1.0433	30.0791	0.9163	0.8905	0.6727	2.0070
^90^Y β	0.3953	84.0775	0.3783	5.1705	0.3354	12.9245
^40^K β	0.7171	69.9045	0.6701	3.1019	0.5448	7.4159
^40^K γ	0.2664	13.1867	0.2340	1.6534	0.1941	5.0102
^137^Cs γ	0.8894	15.2840	0.8031	2.0173	0.5394	6.2769
^226^Ra γ	5.2686	13.9389	4.6326	2.1576	3.0172	7.1939
^214^Pb γ	2.1741	14.9484	1.8676	2.2266	1.2183	7.2108
^214^Bi γ	0.9984	15.2966	0.8905	2.0536	0.5998	6.4716

Each emission case uses 10^6^ histories. Channel C is defined by E_s_ > 18.6 keV for Old and E_l_ ≥ 18.6 keV for the dual diameter layouts. Channels A and C are disjoint within each layout. The entries are not weighted by activity concentration or emission yield.

**Table 8:** Station-based compositions with an actual false-positive probability of at most 5% in the baseline 4 h test.

Layout	Count (Out of 19)	Qualifying Sample IDs	FP in Listed Cases (%)
Old	5/19	S14, S2, S17, S28, S20	4.792–4.976
Inner 5	8/19	S14, S2, S17, S28, S20, S24, S9, S11	4.681–4.991
Inner 10	19/19	S14, S2, S17, S28, S20, S24, S9, S11, S10, S18, S22, S19, S8, S31, S25, S27, S30, S23, H29	4.068–4.599

True tritium concentration is zero, c_e_ = 0.50 and t = 14,400 s. Each layout retains its own L-calibrated κ_0_ for every tested composition. The denominator excludes L and M_ref_; station IDs follow Table 3 and are listed in increasing Cs order. A composition qualifies when its unrounded actual null rejection probability is ≤0.05. The last-column range applies only to the qualifying compositions listed in that row. These are model-based false-positive probabilities under frozen calibration, distinct from composition-correct MDA; passing at 4 h does not guarantee control at every counting time.

**Table 9:** Deposited-energy MDAs in L and H29 at 600 s with composition-specific calibration.

Layout	G/G_Old_	L B_C_ (Counts)	κ in L	L MDA (Bq L^−1^)	H29 MDA (Bq L^−1^)
Old	1.0000	1051.68	0.01048	97.7	105.9
Inner 5	0.8825	48.07	0.21165	120.5	131.8
Inner 7.5	0.7397	73.99	0.12445	131.9	141.5
Inner 10	0.5706	104.26	0.07089	152.9	161.7
Outer 5	0.5839	173.51	0.03927	142.4	153.8
Outer 7.5	0.4353	181.07	0.03050	176.2	188.0
Outer 10	0.3583	178.58	0.02309	193.6	204.4

Each layout uses a simultaneous reference. MDA is evaluated with the correct coefficient for each composition, a nominal α = 0.05 and a detection probability of 0.95. Optical response, residual instrumental background and calibration uncertainty are excluded.

**Table 10:** Comparison of all layouts at c_e_ = 0.75 and 4 h.

Layout	G Retained (%)	Mean RMSE (19 Cases)	H29 RMSE	L MDA	H29 MDA	H29 FP (%)
Old	100.0	11.27	22.72	25.2	27.5	85.32
Inner 5	87.8	11.72	16.12	28.9	31.5	33.26
Inner 7.5	73.6	12.69	14.66	32.3	34.6	14.75
Inner 10	57.8	14.21	15.25	36.7	38.9	7.51
Outer 5	58.9	14.18	21.27	34.4	37.4	45.97
Outer 7.5	44.3	16.30	20.81	41.4	44.6	25.37
Outer 10	36.5	17.39	19.90	44.1	47.2	14.67

RMSE and MDA units: Bq L^−1^. All columns use 14,400 s and identical coupling and PE cuts. The mean gives equal weight to all 19 matched station compositions and does not represent their frequency of occurrence in the environment. RMSE assumes 100 Bq L^−1^ true tritium and frozen L calibration. False-positive probabilities assume zero tritium with the same frozen calibration. Each MDA instead uses its composition-specific correct coefficient. Tritium response is normalized to Old in L. All values are conditional model predictions.

## Data Availability

The computational sample compositions and numerical results supporting the reported comparisons are provided in this article. Sources of the external radionuclide data are identified in the text and reference list.
